# A counseling intervention to address HIV stigma at entry into antenatal care in Tanzania (*Maisha*): study protocol for a pilot randomized controlled trial

**DOI:** 10.1186/s13063-019-3933-z

**Published:** 2019-12-30

**Authors:** Melissa H. Watt, Elizabeth T. Knippler, Linda Minja, Godfrey Kisigo, Brandon A. Knettel, James S. Ngocho, Jenny Renju, Haika Osaki, Rimel Mwamba, Jane J. Rogathi, Blandina T. Mmbaga

**Affiliations:** 1 Department of Population Health Sciences, University of Utah, 295 Chipeta Way, Williams Building, Room 1N410, Salt Lake City, UT 84108 USA; 2 Duke Global Health Institute, Duke University, Box 90519, Durham, NC 27701 USA; 3 Kilimanjaro Clinical Research Institute, Moshi, Tanzania; 4 Kilimanjaro Christian Medical University College, Moshi, Tanzania; 5 Department of Population Health, London School of Hygiene and Tropical Medicine, London, UK; 6 Department of Epidemiology and Biostatistics, Kilimanjaro Christian Medical University College, Moshi, Tanzania; 7 Kilimanjaro Christian Medical Centre, School of Nursing, Moshi, Tanzania

**Keywords:** Tanzania, HIV, Stigma, Intervention, Pilot randomized control trial

## Abstract

**Background:**

HIV-related stigma significantly impacts HIV care engagement, including in prevention of mother-to-child transmission of HIV (PMTCT) programs. *Maisha* is a stigma-based counseling intervention delivered during the first antenatal care (ANC) visit, complementing routine HIV counseling and testing. The goal of *Maisha* is to promote readiness to initiate and sustain treatment among those who are HIV-positive, and to reduce HIV stigmatizing attitudes among those who test negative.

**Methods:**

A pilot randomized control trial will assess the feasibility and acceptability of delivering *Maisha* in a clinical setting, and the potential efficacy of the intervention on HIV care engagement outcomes (for HIV-positive participants) and HIV stigma constructs (for all participants). A total of 1000 women and approximately 700 male partners will be recruited from two study clinics in the Moshi municipality of Tanzania. Participants will be enrolled at their first ANC visit, prior to HIV testing. It is estimated that 50 women (5%) will be identified as HIV-positive. Following consent and a baseline survey, participants will be randomly assigned to either the control (standard of care) or the *Maisha* intervention. The *Maisha* intervention includes a video and counseling session prior to HIV testing, and two additional counseling sessions if the participant tests positive for HIV or has an established HIV diagnosis. A subset of approximately 500 enrolled participants (all HIV-positive participants, and a random selection of HIV-negative participants who have elevated stigma attitude scores) will complete a follow-up assessment at 3 months. Measures will include health outcomes (care engagement, antiretroviral adherence, depression) and HIV stigma outcomes. Quality assurance data will be collected and the feasibility and acceptability of the intervention will be described. Statistical analysis will examine potential differences between conditions in health outcomes and stigma measures, stratified by HIV status.

**Discussion:**

ANC provides a unique and important entry point to address HIV stigma. Interventions are needed to improve retention in PMTCT care and to improve community attitudes toward people living with HIV. Results of the *Maisha* pilot trial will be used to generate parameter estimates and potential ranges of values to estimate power for a full cluster-randomized trial in PMTCT settings, with extended follow-up and enhanced adherence measurement using a biomarker.

**Trial registration:**

ClinicalTrials.gov, NCT03600142. Registered on 25 July 2018.

## Background

Prevention of mother-to-child transmission (PMTCT) programming serves as an essential entry point for HIV testing and linkage to care, and has the potential to eliminate the incidence of vertical mother-to-child transmission. Under the Option B+ guidelines for PMTCT recommended by the World Health Organization, universal HIV testing in antenatal care (ANC) is followed by initiation of lifelong antiretroviral therapy (ART) for pregnant and breastfeeding women living with HIV [1]. Despite the global roll-out of Option B+ programs, retention in HIV care during the pregnancy and postpartum periods has been suboptimal [2, 3].

HIV-related stigma — whether anticipated, internalized, or enacted — has a profound impact on decisions related to HIV [4, 5] and is a primary factor influencing linkage and retention in PMTCT programs [6–8]. In addition, stigma undermines the quality of life of people living with HIV (PLWH), contributing to emotional distress and social alienation [5]. Among HIV-negative individuals, misinformation and prejudicial attitudes about HIV can fuel stigma and contribute to discrimination against PLWH, and can lead individuals to avoid or delay HIV testing [9]. Social environments where enacted stigma occurs, or where stigma is strongly anticipated, contribute to internalized feelings of shame among PLWH and undermine the success of PMTCT programs [10, 11].

Entry into ANC provides a unique opportunity to reach pregnant women and their male partners to reduce community-level HIV stigma and to improve linkage to and retention in HIV care for individuals living with HIV. Addressing HIV stigma at the first ANC visit can help women who test positive to overcome stigma-related barriers to the initiation and maintenance of HIV care, and can help women who know their status to cope with HIV-related stigma during pregnancy and the transition to PMTCT services. Additionally, partner HIV testing during ANC provides a unique opportunity to reach men, in order to address HIV stigmatizing attitudes and improve men’s linkage to HIV care. Long-term HIV care engagement for both women and men has important implications for health outcomes, quality of life, and the risk of future transmission to their child or others.

The goal of this study is to conduct a pilot evaluation of a brief, scalable counseling intervention called *Maisha*, which addresses HIV stigma at entry into ANC. The study will be conducted in Moshi, Tanzania. The intervention includes up to three sessions (one session prior to HIV testing, and two additional sessions for those who test positive for HIV). Male partners will be invited to attend *Maisha* 1 and *Maisha* 2; *Maisha* 3 is for women only. The intervention content is based on principles of cognitive-behavioral therapy (addressing automatic negative thoughts about the self, future, and the world/others) to address and mitigate multiple forms of HIV stigma (internalized, anticipated, and enacted). The intervention approach is novel because it addresses HIV stigma at a key, early, juncture of care time point, and, among those who are HIV-negative, it promotes acceptance and empathy toward PLWH during the heightened emotional period of HIV testing.

## Methods

### Study design

**Fig. 1:**
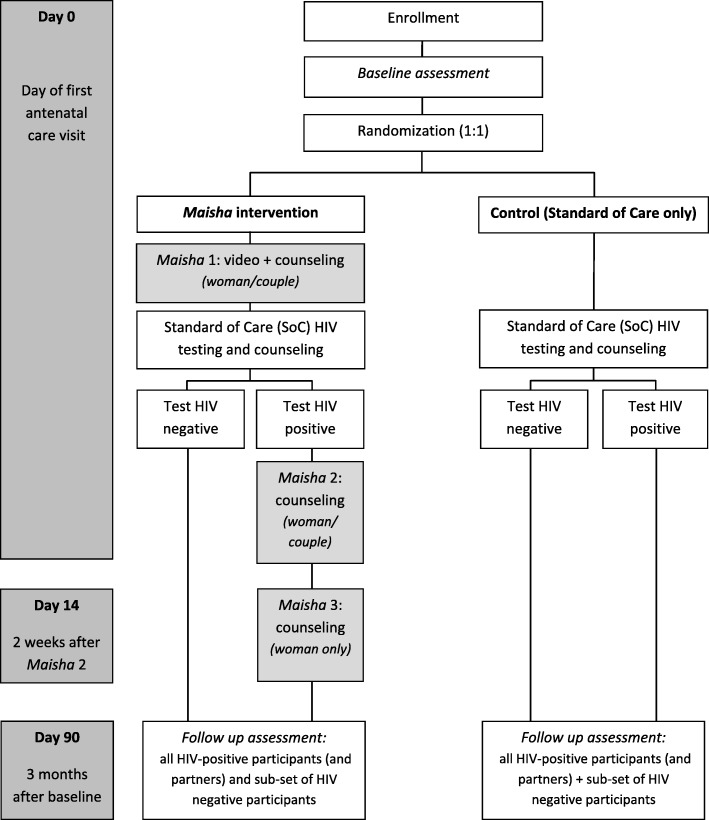
Study flowchart. HIV human immunodeficiency virus

The study is a pilot randomized control trial aimed at determining the feasibility, acceptability, and potential efficacy of an HIV stigma counseling intervention for individuals entering antenatal care (ANC). The study has two parallel groups: control and intervention. The control group will receive standard of care from the clinical sites; the intervention group will receive standard of care paired with up to three *Maisha* counseling sessions. The allocation ratio for these parallel groups is 1:1. Table 1 summarizes key elements of the study, and Fig. 1 illustrates participant flow through the study.

**Table 1:** Study summary (template adapted from the World Health Organization Trial Registration Data Set)

Data category	Information
Title	A Stigma Reduction Intervention at Time of Entry into Antenatal Care to Improve PMTCT Services in Tanzania (*Maisha*)
Primary registry and trial identifying number	ClinicalTrials.gov NCT03600142 Registered July 25, 2018
Secondary identifying numbers	R21 TW011053 (US NIH Grant/Contract)
Primary funder	Fogarty International Center (NIH)
Contact for public queries	Melissa Watt, PhD melissa.watt@utah.edu
Countries of recruitment	Tanzania
Health condition(s) or problem(s) studied	HIV care engagement HIV stigma
Key inclusion and exclusion criteria	Ages eligible: 18 years and older Sexes eligible: all Accepts healthy volunteers: yes Inclusion criteria: 18 years of age or older If female: pregnant and attending first antenatal care (ANC) appointment for the current pregnancy at one of the two study sites If male: accompanying an enrolled woman to her first ANC appointment. Exclusion criteria: Impaired mental status Does not speak Swahili
Study type	Pilot feasibility trial Allocation: randomized Allocation ratio: 1:1 Intervention model: parallel assignment
Date of first enrollment	April 8, 2019
Target sample size	1700 participants
Recruitment status	Recruiting
Primary outcome(s)	HIV care retention (female HIV-infected participants only) (time frame: post assessment (3 months after enrollment)) Internalized HIV stigma (HIV-infected participants only) (time frame: post assessment (3 months after enrollment)) Attitudes toward people living with HIV (HIV-uninfected participants only) (time frame: post assessment (3 months after enrollment))
Key secondary outcomes	ART adherence (HIV-infected participants only) (time frame: post assessment (3 months after enrollment)) Depression (HIV-infected participants only) (time frame: post assessment (3 months after enrollment)) HIV disclosure (HIV-infected participants only) (time frame: post assessment (3 months after enrollment)) Anticipated HIV stigma (all participants) (time frame: post assessment (3 months after enrollment)) Linkage to HIV care (male HIV-infected participants only) (time frame: post assessment (3 months after enrollment)) Willingness to test for HIV in the future (HIV-uninfected participants only) (time frame: post assessment (3 months after enrollment))

*ART* antiretroviral therapy, *HIV* human immunodeficiency virus, *NIH* National Institutes of Health, *PMTCT* prevention of mother-to-child transmission

### Ethical approval and registration

The study has been approved by the ethical review committees at Duke University, Kilimanjaro Christian Medical Center, and the National Institute for Medical Research in Tanzania. The trial is registered at ClinicalTrials.gov (NCT03600142).

### Study setting

The study will be conducted in two government health facilities in Moshi municipality, Tanzania. The Majengo and Pasua Health Centers together see approximately 2500 pregnant women per year; an estimated 4.8% of pregnant women seen at the clinics are living with HIV. All patients are required to have an HIV test at entry to ANC, unless they present a clinic card confirming that they have previously tested positive for HIV. Pregnant patients are strongly encouraged to bring their male partner to the first ANC visit for pregnancy education as well as partner HIV testing.

### Participants

The study will enroll 1000 women into the study. Women will be enrolled at entry into ANC, prior to receiving a routine HIV text; of the 1000 female participants, we expect that approximately 50 will be established or newly diagnosed as HIV-positive. Based on record review at the study clinics, we estimate that 70% of female participants will attend the ANC visit with their male partner, allowing for an enrollment of up to 700 men. The sample size was selected in order to have adequate power to detect differences in our outcome of HIV stigma attitudes for our HIV-negative clients, and to have pilot feasibility data related to outcomes for HIV-positive clients [12, 13].

Participants will be enrolled prior to attending their ANC appointment, which is where routine HIV testing occurs. Following written consent to participate in the study, participants will complete the baseline survey and be randomized to a condition. Following the survey and *Maisha 1* (if randomized to intervention arm), they will return to the clinic for standard of care services, including HIV testing.

Participants will be eligible for the study if they meet the following criteria:

- Minimum 18 years of age
- Women: pregnant and attending first ANC appointment for current pregnancy
- Men: accompanying a partner to her first ANC appointment (note: the partner must be enrolled for the man to be eligible to participate)
- Able to understand and speak Kiswahili
- Able to provide consent

### Procedures

#### Screening and recruitment

First ANC attendees and their partners will be identified in the clinic waiting room by the clinic nurses. The nurses, in cooperation with research staff, will provide a brief description of the research activities, including the time commitment. Individuals who are interested in learning more will meet with the research team in a private research office, either alone or with their partner. The research staff will confirm that the individual(s) meet the eligibility requirements, will clearly explain the study, and will obtain written informed consent. No biological specimens will be collected in this trial; therefore, consent for specimen storage is not applicable.

Contact information will be gathered from participants in order to facilitate the scheduling of follow-up assessments; this information will be securely stored in a locked file cabinet, separate from other participant information and data, and accessed by authorized study staff only.

#### Baseline data collection

After providing consent, all participants will complete a structured survey using audio computer-assisted self-interview (ACASI) technology on tablets running Questionnaire Development System (QDS) software. The ACASI modality can ensure participant privacy and improve data validity by minimizing social desirability bias [14]. Participants will complete the assessment on individual tablets where they can read (on the screen) and listen (through recorded audio) to the questions and response options in Kiswahili. As response options are read aloud, the corresponding text will light up on the screen. Participants can select their response using the tablet touch screen. The computerized assessments are programmed to skip questions that are not applicable based on previous question responses. Validity check items (e.g., “For this question, choose *strongly agree*”) are included throughout to assess for data quality and participant attentiveness. A research staff member will be present in the room to answer any questions and provide assistance as needed. The data files will be securely transferred each day to the local data manager, who will store the files in a centralized location on a secured drive and review the files on a weekly basis for any quality issues. Stored data will be identified by a unique ID only, with access limited to authorized investigators and staff.

#### Allocation

Upon completion of the baseline assessment, the participant will be randomized to receive either the standard of care HIV testing and counseling or the standard of care plus the *Maisha* intervention. Female participants will be randomized at a 1:1 ratio using a block randomization method (10 per block) to ensure equal sample sizes by condition and to manage the flow of participants to the intervention condition. The allocation sequences will be prepared ahead of time by a statistician using an online randomization program (www.sealedenvelope.com). Sequences will be generated separately for each of the two study clinics. Study staff who are not involved in participant enrollment, assignment, assessment, or delivery of the intervention will prepare sealed, opaque envelopes for each study ID containing the randomized condition assignment. After a female participant completes the baseline assessment, she will open the envelope marked with her corresponding study ID and will learn her assignment; male participants will be assigned to the same condition as their partners. Participants will not be blinded to their allocation, as all participants will be aware of the additional time and activity commitments required as part of participation in the intervention condition. Since the trial is unblinded, emergency unblinding is not applicable. The research staff who give the participants their assignment envelopes will not know the randomization sequence until the conditions are assigned. Health care personnel at the study clinics will not be informed of participants’ study conditions, in order to prevent interference in standard of care delivery.

#### Experimental conditions

##### Control: standard of care HIV testing and counseling

Participants randomized to the control condition will receive the standard HIV testing and counseling protocol in the clinic, which is administered by clinic nurses. The standard of care was chosen as the comparator in order to evaluate whether the *Maisha* intervention has an impact above and beyond the standard clinic procedures for HIV counseling and testing. According to the Tanzania PMTCT guidelines, HIV pretest counseling should provide education about HIV and prepare a woman (and her partner, if present) for HIV testing [15]. For anyone who tests positive for HIV, counseling should help the woman/couple to accept an HIV test result and discuss implications for treatment. HIV-infected women should be registered for PMTCT and immediately initiated on ARVs. HIV-infected men should be referred to the HIV care and treatment clinic (CTC) for same-day initiation of ARVs.

##### Intervention: standard of care and *Maisha*

Participants randomized to the intervention condition will attend the standard of care HIV testing and counseling, and will also receive the *Maisha* intervention. *Maisha* is a brief, scalable, theory-based counseling intervention that addresses HIV stigma at entry into antenatal care (ANC) and includes up to three counseling sessions. The intervention model combines a stigma framework with principles of cognitive-behavioral therapy to address and mitigate the impact of stigma on health outcomes. In Earnshaw and Chaudoir’s HIV Stigma Framework, internalized, anticipated, and enacted stigma all intersect to undermine health-seeking behaviors [4, 16]. In developing the intervention framework, we observed that these components of stigma track onto the CBT “cognitive triad” of negative beliefs about oneself, the future, and others/the world [17]. Thus, the *Maisha* intervention addresses these three forms of stigma using principles of cognitive-behavioral therapy and formative work by Tshabalala and Visser [16, 18].

**Fig. 2:**
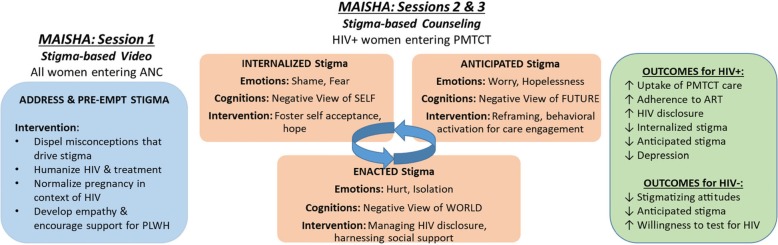
Intervention theoretical framework. ANC antenatal care, ART antiretroviral therapy, HIV human immunodeficiency virus, PLWH people living with HIV/AIDS, PMTCT prevention of mother-to-child transmission

Upon entry to ANC (for most, prior to learning their HIV status), we will deliver information, present the lived experiences of PLWH using a video, and promote self-reflection about community attitudes related to HIV. Participants will examine how community perceptions influence their beliefs about PLWH, including prejudicial beliefs that contribute to self-stigma in the event of a positive test. In *Maisha* 2 and 3, with participants who are HIV-positive (either presenting to ANC knowing their status or getting a new diagnosis of HIV), we will review the video content and provide additional structured counseling to address the difficult emotions and cognitions often associated with a positive status. Through linkage to the video, we will use Beck’s interventions for cognitive bias [19, 20] as well as Third Wave behavioral concepts [21, 22] to address one’s automatic negative thoughts about the self (internalized HIV stigma), the future (anticipated HIV stigma), and the world (enacted stigma). Intervention techniques include recognizing and reframing cognitive distortions, helping individuals to develop a positive self-schema, and promoting personal acceptance and value-driven behavior to reduce stigma and encourage positive HIV care engagement (Fig. 2). Table 2 provides an overview of the *Maisha* intervention.

**Table 2:** Overview of the *Maisha* intervention

Session information	Content	Goals
*Maisha* 1 Population: all intervention participants (women only or couples); separate guides for participants with unknown HIV status and participants with known HIV diagnosis Timing: before standard of care ANC visit	Watch 8-min video telling the story of Salma and Bahati, a couple who test for HIV at their antenatal care visit and learn how to navigate their diagnosis Review video and discuss topics related to both Salma and participant: • Feelings during HIV testing • Thoughts about the future • Anxieties related to HIV testing • Importance of HIV care engagement • Supportive individuals Introduce and discuss the three types of stigma: internalized, enacted, anticipated Final messages • If you test positive, there are medications available and people who can support you • If you test negative, you can be a source of support for other people who have HIV For participants with a known HIV diagnosis, the session involves a discussion of how the video and types of stigma relate to the participants’ own experiences of living with HIV	• Normalize HIV and increase empathy for people living with HIV • Raise consciousness regarding HIV stigma, and rethink stigmatizing attitudes • Prepare participants for HIV testing and acceptance of a possible HIV diagnosis
*Maisha* 2 Population: all intervention participants with an HIV diagnosis (couples attend if at least one person in the couple has an HIV diagnosis); separate guides for new HIV diagnoses and established HIV diagnoses Timing: same day as *Maisha* 1, after ANC visit	Link back to video to provide hope for the future and address the three types of HIV stigma: Internalized stigma • Acknowledge negative emotions • Reassure about accepting one’s HIV status with time Anticipated stigma • Acknowledge worries • Reassure about their future Enacted stigma • Acknowledge that it may take time to disclose and get support Identify values and link to adherence/care engagement Final messages • Acceptance is a process that takes time • Your values can help you commit to taking treatment and attending the clinic	• Address immediate stigma-related concerns and provide reassurance • Create commitment to treatment and a plan to return to the same clinic for the next HIV appointment
*Maisha* 3 Population: all HIV-infected female intervention participants (women only); separate guides for new HIV diagnoses and established HIV diagnoses Timing: 2 weeks after *Maisha* 2	Link back to video to help the client develop an action plan, addressing the three types of stigma: Internalized stigma • Discuss feelings about oneself as someone living with HIV • Action plan for how one can come to accept self as someone living with HIV Anticipated stigma • Discuss worries related to attending the clinic (especially related to others learning about one’s HIV status) • Action plan for attending clinic and taking ARVs Enacted stigma • Discuss any disclosures and support • Action plan for disclosing and/or harnessing support to stay in care Discuss HIV and personal care, including: • Check-in on taking medication • Establish connections between thoughts and feelings using a CBT model; discuss coping mechanisms • Introduce a mindfulness/breathing exercise • Discuss challenges and make a commitment to care Final messages • Just taking the step to be here at this session and at the clinic is an important one and something to be proud of • Acknowledging and addressing worries can help us stay positive and find support • It is important to keep coming to care and taking medications	• Build on the previous sessions to prevent or reduce internalized and anticipated stigma • Develop strategies to cope with or mitigate enacted stigma, while getting support • Develop commitment to PMTCT care, and create a plan for overcoming barriers to care

*ANC* antenatal care, *ARV* antiretroviral, *CBT* cognitive-behavioral therapy, *HIV* human immunodeficiency virus, *PMTCT* prevention of mother-to-child transmission

The development of the *Maisha* intervention was informed by our team’s previous research [2, 3, 23], qualitative interviews with patients and health care providers, and input from a study advisory board. In order to develop the video content, we engaged the local community advisory group, comprising individuals who were living with or affected by HIV. These “expert patients” helped us to develop the video, which tells the story of a pregnant woman and her husband who both test positive for HIV and take steps to cope with the diagnosis. The actors for the video were selected from the community advisory group, and participated in the iterative refinement of the script during filming. Following the production of the video and the development of the intervention scripts, we conducted a trial run of the intervention with eight participants recruited from the ANC. Their input was elicited, which contributed to further modifications in the final intervention.

The *Maisha* intervention will be delivered by Bachelor’s-level counselors who have social work or counseling backgrounds. Counselors will receive a minimum of 2 weeks of training on counseling techniques and the intervention content, and thereafter will receive at least 1 h per week of clinical supervision, with the opportunity for additional supervision as needed. The counseling sessions will take place in a private research office at the clinic site.

##### Intervention fidelity

The intervention counselor will complete a quality assurance (QA) and process rating form after each session. This form records issues raised in the session, coverage of session content, and feasibility of delivery. All intervention sessions will be recorded (with participant consent), and each week 1 session from each counselor will be reviewed during a group supervision session. Using a structured form [24], the counselors and supervising staff will assess the recorded sessions for intervention fidelity and presence of core components of counseling. During the group supervision session, the team will review the recording to discuss challenges and provide additional feedback and training.

##### Scheduling

For participants who are assigned to receive *Maisha* 2 and *Maisha* 3, the counselor will aim to deliver *Maisha* 2 on the same day as enrollment and *Maisha* 1 (i.e., the day of the first ANC appointment). If this is not possible (e.g., if the participant does not return after testing, or the participant does not have time to complete the session that day), the counselor will attempt to schedule the session for another day, ideally within 72 h. *Maisha* 3 will be scheduled approximately 2 weeks after completing *Maisha* 2. In order to reduce the burden on participants and improve attendance, the counselor will try to schedule *Maisha* 3 on the same day as the participant’s next clinic appointment. The counselor will call participants to remind them of upcoming sessions. If a participant misses a scheduled session, the counselor will call to follow-up and reschedule if possible. Participants may choose to stop attending *Maisha* sessions or withdraw from the study at any time.

During the study period, participants will continue with the standard antenatal care, which includes routine HIV pretest and posttest counselling for unknown status. The intervention is intended to supplement, not replace, existing clinical care, and therefore does not impact use of or access to routine medical care.

#### Follow-up assessment

A subset of participants (approximately 300 women and 200 men) will be selected to complete a follow-up assessment 3 months after completing the baseline assessment. All HIV-positive participants and their partners (if enrolled) will be contacted for follow-up. A subset of HIV-negative participants will also be eligible for follow-up. In order to observe changes in stigmatizing attitude scores among HIV-uninfected participants, individuals with stigmatizing attitude scores greater than 14 will be eligible for follow-up assessment; of those who meet the criteria, a random 60% will be invited for follow-up.

Participants selected for follow-up will complete a structured post assessment, following the same procedures for ACASI-based data collection used for the baseline assessment. At the end of the assessment, all *Maisha* intervention participants will also respond to a short series of open-ended feedback questions about the intervention; this section of the assessment will be orally administered and audio-recorded to fully capture participant responses. Responses to the open-ended questions will be directly translated into English from the audio-recording, and entered into a REDCap database. For all HIV-positive study participants, data on HIV care engagement will be abstracted from their medical records and entered into REDCap using double data entry, allowing for data quality checks and secure data storage and transfer.

#### Participant tracking and retention

Research staff will conduct a daily review of the clinic ANC logs to record the number of potentially eligible participants who were not enrolled in the study. For enrolled participants, the HIV test results and estimated date of delivery will be recorded on the day of enrollment. The research staff will maintain tracking logs to record the dates when assessments and *Maisha* sessions are scheduled and completed. In preparation for the scheduled sessions, research staff will contact participants to remind them of upcoming appointments in order to ensure retention. If participants are unreachable or did not provide a contact number, research staff will consult the participant’s medical record for upcoming appointments and try to speak to them in person to schedule a follow-up visit. All participants will receive a transport allowance (5000 TSh = approximately 2 USD) to facilitate their return to the clinic. For participants who fail to return for a scheduled follow-up appointment, the reasons will be documented and collated across participants.

### Outcomes

Measures were selected based on previous research in East Africa, including measure validation when available, and evaluation of face validity by the Tanzanian researchers on our team. All measures were translated from English into Swahili and then back-translated and discussed to reach consensus on best translation. Table 3 summarizes the outcome measures that will be assessed at baseline and 3-month follow-up, specific to the participant’s known HIV status.

**Table 3:** Outcome measures

	Baseline*	Post assessment (3 months)	
	HIV unknown	HIV-positive	HIV-negative
Primary outcomes			
HIV-infected study participants			
HIV care engagement		X	
Internalized stigma		X	
HIV-uninfected study participants			
Attitudes toward PLWH	X		X
Secondary outcomes			
HIV-infected study participants			
ART adherence		X	
Depression (EPDS/PHQ-9)	X	X	
HIV disclosure		X	
Anticipated HIV stigma	X	X	
Linkage to care (male participants)		X	
HIV-uninfected study participants			
Willingness to test for HIV in the future	X		X
Anticipated HIV stigma	X		X

*ART* antiretroviral therapy, *EPDS* Edinburgh Postnatal Depression Scale, *HIV* human immunodeficiency virus, *PHQ-9* Patient Health Questionnaire, *PLWH* people living with HIV/AIDS

*For women who present to antenatal care as known HIV infected, the following measures will also be done at baseline: HIV care engagement, adherence, internalized stigma, and HIV disclosure

#### Primary outcomes for HIV-positive participants

##### HIV care retention (women)

Among female HIV-positive participants, retention in care at 3-month follow-up will be assessed via medical record review, with retention defined as having no more than a 60-day gap between PMTCT visits at the study clinic or having a record of an official transfer to another clinic [25]. We will assess differences between conditions in the proportion retained in care at 3 months.

##### Internalized HIV stigma

Among HIV-infected participants, internalized stigma will be self-reported, measured by Scale A of the HIV and Abuse Related Shame Inventory (HARSI) [26] plus one added item. Internalized stigma will be measured at baseline (among individuals with established HIV diagnoses) and 3 months (among individuals with new and established HIV diagnoses). We will assess differences between conditions in mean scores of internalized HIV stigma scores at 3 months. For individuals with established HIV diagnosis, we will control for baseline scores.

#### Primary outcomes for HIV-negative participants

##### Attitudes toward people living with HIV (PLWH)

Attitudes will be measured by self-report, using a modified version of the Personal and Attributed Stigma Scale (PASS) which includes two subscales: blame/judgment and interpersonal distancing [27, 28]. The scale was adapted to the local context based on formative qualitative data collection and revised after a pilot of the measure with 88 individuals. Attitudes will be measured at baseline and 3 months; individuals with a known HIV diagnosis will not be assessed on their attitudes toward PLWH. We will assess differences between conditions in mean attitudes scores at 3 months, controlling for baseline scores.

#### Secondary outcomes for HIV-positive participants

##### ART adherence

Adherence to antiretroviral therapy (ART) at the 3-month follow-up will be measured by self-reported medication adherence; missing two or more pills in the past 30 days (< 94% adherence) will be considered poor adherence. We will assess differences between conditions in the proportion with poor adherence at 3 months.

##### Depression

Depression will be measured by the Edinburgh Postnatal Depression Scale (EPDS) [29] for women and the Patient Health Questionnaire (PHQ-9) for male partners [30]. Depression will be measured at baseline and 3 months. We will assess differences between conditions in mean attitudes scores at 3 months, controlling for baseline scores.

##### HIV disclosure

HIV disclosure will be measured by self-report of whether participants have ever disclosed their HIV status to a person outside the heath care workers directly involved in their antenatal and PMTCT care. HIV disclosure will be measured at baseline (among individuals with established HIV diagnoses) and 3 months (among individuals with new and established HIV diagnoses). We will assess differences between conditions in the proportion with poor adherence at 3 months. For individuals with an established HIV diagnosis, we will assess differences in the change in proportion of participants who have disclosed their HIV status in each condition between baseline and 3 months.

##### Anticipated HIV stigma

For HIV-positive participants, anticipated HIV stigma will be measured using an adapted scale [31], which includes 16 items assessing the degree to which PLWH expect they would experience prejudice and discrimination from others if their status was known. Anticipated stigma will be measured at baseline (among individuals with established HIV diagnoses) and 3 months (among individuals with new and established HIV diagnoses). We will assess differences between conditions in mean scores of anticipated HIV stigma scores at 3 months. For individuals with established HIV diagnosis, we will control for baseline scores.

##### Linkage to care (men)

Among HIV-positive male partners, linkage to care at a care and treatment clinic (CTC) will be self-reported at the 3-month follow-up, with linkage to care defined as having attended any CTC appointment. We will measure differences between conditions in the proportion of HIV-positive men attending a CTC for HIV care at 3 months.

#### Secondary outcomes for HIV-negative participants

##### Willingness to test for HIV in the future

Willingness to test for HIV in the future will be measured by self-report of whether participants intend to test for HIV in the next 12 months. Willingness to test for HIV will be measured at baseline and 3 months. We will assess differences in the change in proportion of participants who have disclosed their HIV status in each condition between baseline and 3 months.

##### Anticipated HIV stigma

For HIV-negative participants, anticipated HIV stigma will be measured using an adapted scale [31], which includes 16 items assessing the degree to which participants would expect to experience prejudice and discrimination if they were to be told they were living with HIV. Anticipated stigma will be measured at baseline and 3 months. We will assess differences between conditions in mean scores of anticipated HIV stigma scores at 3 months, controlling for baseline scores.

#### Quality assurance (QA) data

Participant satisfaction with the intervention will be assessed at 3-month follow-up via structured and open-ended questions about satisfaction with the intervention and facilitator, satisfaction with the timing and length of the sessions, ability of the intervention to address issues specific to participant’s experience and context, and suggested changes to the intervention. Intervention sessions will be recorded and a subset of recordings will be reviewed to assess whether core components of the sessions were completed, and to evaluate the effectiveness of the counselor in achieving session objectives.

### Statistical analysis

#### Feasibility, acceptability, and potential efficacy of the intervention

Data analysis will follow guidelines of the CONSORT 2010 statement, as extended to pilot feasibility trials [32]. Feasibility and acceptability of the intervention and the associated trial will be described by recruitment and retention patterns, participant satisfaction, and fidelity of intervention delivery. Retention will be monitored to calculate the percentage of eligible intervention participants who attend *Maisha* 2 and *Maisha* 3 and participants who complete the 3-month assessment. The team will document barriers to attendance for participants and will examine differences between participants who attend and those who do not. Participant satisfaction data at the 3-month follow-up will be described, with > 80% satisfaction used as a metric of acceptability. Open-ended questions will be thematically coded to summarize participants’ perceptions of the intervention, suggestions for changes, and feasibility moving forward. The fidelity to the intervention will be assessed by examining the percentage of components from the *Maisha* session guides that are covered in each session.

Potential efficacy will be examined by analyzing separate outcomes for HIV-positive and HIV-negative individuals. For HIV-positive individuals, we are interested in differences between conditions in health outcomes (retention in PMTCT and medication adherence for women, linkage to CTC for men, and depression) and stigma constructs (anticipated stigma, internalized stigma, and HIV disclosure). For HIV-negative individuals, we are interested in differences between conditions in stigma constructs (attitudes to PLWH and anticipated stigma) as well as willingness to test for HIV in the future. Additional analyses will examine changes in stigma attitudes by subscales (i.e., moral judgment and social distancing) and will include stratified analysis by gender.

For outcomes where there is a baseline measure, mixed-effects regression analysis will be used to model pre–post differences within and between arms, using a time by condition model specification (time, condition, and time × condition). Individual-level random intercepts will be used to account for correlation due to repeated measurement. Using a mixed-effects regression approach leaves flexibility to control for baseline outcome values that may not be balanced between groups due to small sample size, and may improve precision of treatment effect estimation. For outcomes where there is no baseline measure, we will examine differences in means or proportions with 95% confidence intervals. If we suspect, a priori, that baseline imbalance in prognostic covariates may be an issue, we will move into a regression framework. Given ACASI data collection methods, we expect low amounts of missing data. In cases of missing data, multiple imputation methods will be used.

Analysis of outcomes for the HIV-negative participants will be completed using a subsample of enrolled participants who are selected to complete a follow-up survey. Primary analysis will be done on an intention-to-treat (ITT) basis including all cases who were selected for a follow-up survey. We also conduct secondary analysis on a per-protocol (PP) basis considering only participants who received all intervention sessions and completed follow-up. Furthermore, sensitivity analysis will be conducted to assess the effect of missing data on results. Some interim analysis will be performed to check study progress and monitor for adverse events. For clients who are HIV seropositive, apart from retention in care, impact on other outcomes (e.g., internalized HIV stigma) will be assessed only among clients who completed the baseline survey knowing their HIV status.

### Monitoring

Given the low-risk nature of the counseling intervention, we do not anticipate any adverse events as a result of intervention exposure. Therefore, we will not have an independent data monitoring committee. The study team will monitor adherence to the protocol and will report any potential adverse events to the institutional review boards. To ensure the quality of our science, we will have daily check-ins with the field team to monitor daily enrollment of clients, challenges or issues with the consenting process, and any matters arising during survey administration and intervention delivery. The data manager will do a thorough review of the data on a weekly basis, and queries will be sent to research assistants for clarification. Any pressing issues in need of discussion or decision-making will be discussed with the broader team during a weekly team call. Call minutes will be documented and shared with the full team. Due to the low likelihood of adverse effects as a consequence of trial procedures, post-trial administration of counselling is not applicable.

The depression measure that is included in the survey includes a question that assesses for suicidal ideation [29, 30]. If the suicidal ideation question is endorsed, a coded message will be displayed to the research staff at the end of the assessment. The counselor will also make note if a participant says something during a *Maisha* session that indicates they are experiencing suicidal ideation. In either of these cases where ideation is indicated, the staff will follow a protocol to assess the risk, identify sources of support, make a plan for the participant’s safety, and make referrals to clinic personnel as needed. Research staff have been trained on procedures related to emotional distress and the assessment of personal safety and risk, and will report any adverse events to the study coordinator and the local Principal Investigator for further follow-up.

### Dissemination

A study advisory board has been established to provide ongoing stakeholder input on the study and share emerging data and findings. The board will be convened for three half-day workshops during the study: initially, for input on intervention content; mid-way, for feedback on the curriculum and preliminary findings; and at the conclusion, for interpretation/dissemination of results. Advisory board members will be sent a quarterly newsletter with updates on the study progress.

At the conclusion of the study, we will conduct a feedback forum with a larger audience of stakeholders from a variety of institutions, including clinic staff and patients, regional representatives of the Ministry of Health, HIV advocacy and service organizations, and women’s health organizations. During the forum, the team will share the findings of the study and facilitate a discussion about the implications of the data for future research and practice. Results will also be published in peer-reviewed journals and presented at appropriate scientific meetings, including regional, national, and international meetings. Authorship eligibility guidelines will follow the authorship guidelines of the International Committee for Medical Journal Editors (www.icmje.org); we do not intend to use professional writers in the reporting of study results.

All study investigators, along with the data management team, will have access to the final trial dataset. The observational data may be analyzed to answer research questions beyond our stated objectives, and researchers from outside the team can request access to the data. Data can be shared with a data transfer agreement from the Tanzanian National Health Research Ethics Review Committee and within the constraints required for the protection of confidentiality for study subjects. Shared data will not include identifiable information.

### Study organization

As the Principal Investigators of the Maisha intervention, MHW and BTM are charged with co-leading the study. They will ensure the completion and integrity of the study by managing and monitoring study activities and the reporting of study findings. They will facilitate collaboration between Duke University and KCMC by initiating and maintaining communication between these two institutions and the study staff at both locations. MHW and BTM will monitor the ethical overall conduct of research activities, and be responsible for overseeing compliance of financial expenditures in accordance with sponsoring agency regulations.

The faculty investigators in the study, JSN and JR, will bring expertise on PMTCT care delivery and mental health to the *Maisha* intervention. They will support the scientific oversight of the study, meeting weekly with study staff and providing ongoing supervision and support.

A minimum of one data collection staff member will be based at each of the clinical sites, and be responsible for recruiting participants and obtaining study data through surveys (using ACASI technology) and qualitative interviews. One counselor will be based at each clinical site, and will be responsible for delivering the *Maisha* sessions. The data management team, led by statistician LM at KCMC, will be responsible for storing, analyzing, and interpreting quantitative data. The team will clean data and code measures at each time point in order to ensure that the data are valid and easily interpreted.

To elicit stakeholder input, we have established a study advisory board (see “Dissemination”) that includes representatives from the Tanzanian Ministry of Health, leadership in the study clinics, community-based organizations, and members from the KCMC HIV Community Advisory Board.

## Discussion

Despite the significant impact of stigma on HIV care engagement, few evidence-based interventions to address HIV stigma exist [33–35]. The *Maisha* intervention is novel because it addresses HIV stigma during the first ANC appointment, which is a key, early juncture of HIV care for women who test positive. Additionally, it takes advantage of universal HIV testing of women and their partners during the first ANC visit, and promotes acceptance and empathy toward PLWH during the heightened emotional period of HIV testing.

Although this study includes a large number of participants (*n* = 1700), it remains a pilot feasibility trial because the estimated number of HIV-positive participants (*n* = 50 women) remains underpowered to evaluate the primary outcome of retention in PMTCT care. If the preliminary data demonstrate that the intervention is feasible and acceptable, with potential to impact our study outcomes, we will move forward with a full cluster-randomized trial of the intervention in an increased geographical area within the PMTCT setting. A future study will have a larger sample size to capture more HIV-positive women and include longer follow-up through the postpartum period, and we will use biomarker outcomes to assess medication adherence and HIV progression.

The *Maisha* trial was designed with consideration for future scale-up and implementation in the Tanzanian setting. This includes efforts to use existing clinic resources, minimize additional costs, and avoid placing burden on thinly stretched clinic providers. In this pilot feasibility trial, we will examine the potential for integrating *Maisha* into routine clinical care as a way to address community-level HIV stigma and promote HIV care engagement. We will also assess the feasibility of delivery by Bachelor-level counselors, and examine whether the video format of *Maisha* 1 will allow for efficient and standardized delivery of the session content. Future iterations of the intervention may seek to train existing clinic staff to deliver *Maisha* as an enhancement to the standard of care, and to deliver the video content in a group format within the clinic space. Should *Maisha* prove feasible and acceptable in our feasibility trial, we will engage a broader group of stakeholders and policy-makers to explore future scale-up.

## Trial status

This trial was registered at ClinicalTrials.gov (NCT03600142) on 25 July 2018. The first participant was enrolled on 8 April 2019. Participant recruitment and enrollment is ongoing and expected to be completed by March 2020, with final follow-up expected by June 2020.

## Supplementary information

**Additional file 1.** SPIRIT 2013 Checklist: Submitted for manuscript A counseling intervention to address HIV stigma at entry into antenatal care in Tanzania (*Maisha*): Study protocol for a pilot randomized controlled trial.

## Data Availability

Data can be shared with a data transfer agreement from the Tanzanian National Health Research Ethics Review Committee and within the constraints required for the protection of confidentiality for study subjects.

## Abbreviations

ACASI: Audio computer-assisted self-interview
ANC: Antenatal care
ART: Antiretroviral therapy
ARV: Antiretroviral
CTC: Care and treatment clinic
HIV: Human immunodeficiency virus
PLWH: People living with HIV/AIDS
PMTCT: Prevention of mother-to-child transmission
QA: Quality assurance
QDS: Questionnaire Development System
